# Thermal and UV Hydrosilylation of Alcohol-Based Bifunctional Alkynes on Si (111) surfaces: How surface radicals influence surface bond formation

**DOI:** 10.1038/srep11299

**Published:** 2015-06-12

**Authors:** Y. L. Khung, S. H. Ngalim, A. Scaccabarozi, D. Narducci

**Affiliations:** 1 University of Milan-Bicocca, Department of Materials Science Via R. Cozzi 55, I-20125 Milan (Italy); 2 Regenerative Medicine Cluster, Advanced Medical and Dental Institute (AMDI) Universiti Sains Malaysia, Penang (Malaysia)

## Abstract

Using two different hydrosilylation methods, low temperature thermal and UV initiation, silicon (111) hydrogenated surfaces were functionalized in presence of an OH-terminated alkyne, a CF_3_-terminated alkyne and a mixed equimolar ratio of the two alkynes. XPS studies revealed that in the absence of premeditated surface radical through low temperature hydrosilylation, the surface grafting proceeded to form a Si-O-C linkage via nucleophilic reaction through the OH group of the alkyne. This led to a small increase in surface roughness as well as an increase in hydrophobicity and this effect was attributed to the surficial etching of silicon to form nanosize pores (~1–3 nm) by residual water/oxygen as a result of changes to surface polarity from the grafting. Furthermore in the radical-free thermal environment, a mix in equimolar of these two short alkynes can achieve a high contact angle of ~102°, comparable to long alkyl chains grafting reported in literature although surface roughness was relatively mild (rms = ~1 nm). On the other hand, UV initiation on silicon totally reversed the chemical linkages to predominantly Si-C without further compromising the surface roughness, highlighting the importance of surface radicals determining the reactivity of the silicon surface to the selected alkynes.

Grafting organic monolayers on silicon surfaces often require bifunctional or multifunctional molecules that have one end adherent to the surface while having the other active distal ends exposed so as to permit for further modifications. This multiple functionality is deemed necessary to graft useful organic monolayers on silicon surfaces, especially in biological applications^1^,^2^. Currently, attaching organic films covalently onto silicon surface is most commonly achieved through Silane chemistry although there are some shortcomings to its uses^3^,^4^. Firstly, the control of film thickness can be difficult and highly unpredictable due to the cross-linking nature of the film. This alone presents a major challenge in devices demanding stringent precision in the height of monolayers on surfaces, i.e. electronic-based biosensors etc., in order to reduce the problem of Debye screening^5^,^6^. There is also an issue of silanol bond degradation over time in aqueous conditions^7^ that in turn further impedes its use outside the laboratory setup.

In light of the above challenges, many research groups had opted for the more stable Si-C linkage for monolayer assembly on silicon surface, typically attainable through the hydrosilylation chemistry route. The reaction can be typically described as a Flory-like propagation on the surface that gets initiated with the deliberate radicalization on the surface via the ejection of hydrogen from silicon hydrogenated surface (Si-H), thus producing an on-site reactive silyl radical^8^,^9^,^10^. This surface radicals are highly reactive towards unsaturated carbon bond as they can be transferred to the alkyl chain and subsequently back to adjacent silicon atom which, in the process, abstracts another hydrogen to form another radical and the sequence of events repeats itself. The result would be a self-terminating propagation of a Si-C linked organic monolayer that is highly homogenous in height and density.

So far, Si-C linked organic monolayers were reported to be more stable than those produced from silane chemistry, especially in aqueous conditions^11^,^12^. The fact that the absence of cross-linking between molecular chains would mean that the film thickness can be very easily determined and controlled and monolayer grafting can truly be attained. Currently, there is a wide range of methods describing the formation of stable Si-C on hydrogen-terminated silicon^8^,^13^,^14^. Regardless of the approach, the standpoint had always been the same, i.e. the abstraction of the hydrogen atom from Si-H surface to form a radical point on the surface at the first stages and this is deemed as an essential first step to initiate the reaction. As such, much of the emphasis has often been in energy reduction in the first step to radicalize the surface. This had brought about the popular use of transition metals based catalytic agents and other organic intermediaries^15^,^16^. The major drawback in using such catalyst during hydrosilylation is the residual leftover of the metallic catalysts are often difficult to displace with normal cleaning methods, and they may interfere with the silicon surface properties. This had evidently opened other avenues and opportunities for non-catalytic hydrosilylation approaches such as UV initiation and thermal based hydrosilylation. UV initiation, as its name suggest, involves subjecting the silicon surface to an initial high dosage of UV irradiation to introduce radical on the surface^17^,^18^,^19^ while thermal hydrosilylation works on using high temperature to achieve the same outcome^20^,^21^. Both techniques are very popular although they are not without their drawbacks. UV-initiation of the surface can be highly susceptible to surface oxidation or contamination from residual oxygen or water while the process of thermal hydrosilylation can be relatively lengthy (up to days) and require temperatures above 150 degrees to achieve surface radicalization. Nonetheless, the attraction with thermal hydrosilylation and UV hydrosilylation remain high due to many reports showing good quality films being produced. Interestingly, in recent years, there had been reports suggesting that at temperature lower than 150 °C, the surface can still undergo hydrosilylation. This reason was proposed by Horrocks *et al.* in mid-2000s as the abstraction of hydrogen via trace amount of oxygen in solution can form a viable radical site^22^. This had further increased the appeal of performing thermal hydrosilylation at temperatures below 150 °C.

For bifunctional alkyne molecules to be thermally grafted to Si-H surface via the Si-C linkage, it is important to pay special attention to the end group type as there had been several reports highlighting the possibility of -OH reacting to the surface to form Si-O-C linking chemistry even in mild UV irradiation as demonstrated by Hacker *et al.*^23^,^24^. Although there are a number of studies reporting on the formation of Si-O-C linkage either using alcohol or aldehyde terminus group^23^, there is little comparison in literature pertaining to how these functional groups may interfere with the unsaturated carbon distal ends in the classical hydrosilylation reaction. Previously our group had already published a report highlighting the susceptibility of Si-H surface undergoing nucleophilic reactions at lower temperatures (130 °C) with OH terminated alkynes to form Si-O-C linkages onto the surface instead of the Si-C bond driven by the alkyne ends even though alkynes had been reported to be extremely reactive towards the Si-H surface^21^. This was interesting as our reaction was performed at temperatures that rendered the surface ‘radical-free’ at the initial stages (less than 150 °C). However, whether a surface pre-decorated with radicals may influence the outcome of the OH terminated alkyne reacting to the surface remains relatively unknown.

Hence, in this paper, we examine the effect arising from the presence or absence of radicals on Si-H surface influencing its reactivity outcome to short aromatic bifunctional alkynes, akin to the phenylacetylene species as examined by Buriak *et al.* previously^25^,^26^, with the primary focus directed towards the formational chemistry of surface bonds. This was achieved by performing two different methodologies for hydrosilylation, low temperature thermal hydrosilylation and UV-initiated hydrosilylation. To study the underlying reactivity, two different bifunctional alkynes were selected, a CF_3_ terminated alkyne (to raise the hydrophobicity profile) and an OH terminated alkyne (to increase the wettability). UV initiated hydrosilylation would produce the silicon surface with radicals for reaction to the bifunctional alkyne while low temperature hydrosilylation was performed in conjunction as a comparative means to produce a surface without pre-existing radicals. We sought to follow and establish the role by which surface radicals at the initial stages plays in response to a bifunctional alkyne that can potentially react on both ends, either at the CH end to form a Si-C linkage or via the OH end to form Si-O-C type linkage. Next, we also implemented the hydrosilylation reaction in equimolar concentration of both alkyne molecules in order to further extricate any underlying preferential behaviour or competition for reactivity. Finally, we examined the surface wettability and topographical profile arising from these surface reactivity changes.

## Results and discussion

**Figure 1:**
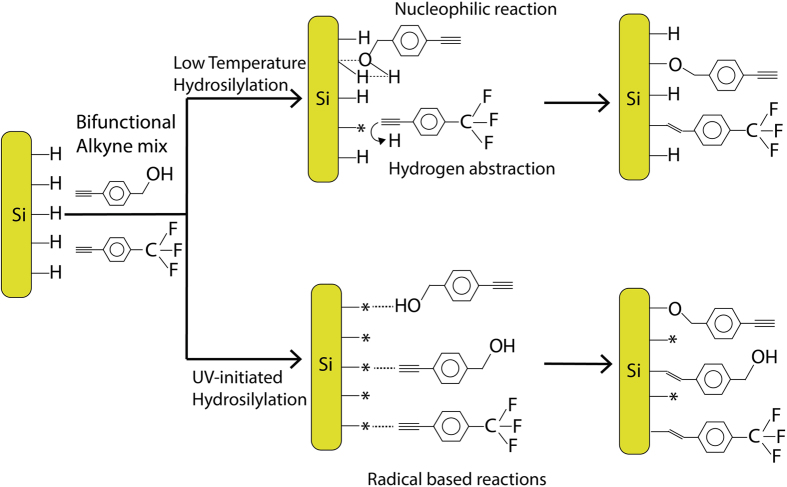


Based on our previous study^21^, our current hypothesis is that in the absence of radicals, Si-H surface is very prone to nucleophilic reaction in the presence of OH species, thus forming a Si-O-C type linkage while in the absence of OH species, the radical-free surface can still react to the alkyne to form Si-C linkage using the hydrogen abstract concept proposed by Horrocks *et al.*^27^. However when the surface is pre-decorated with radicals, the reaction should be deemed indiscriminate towards either OH or CH end in the bifunctional alkyne, thus ensuring that both ends were anchored to form a ‘mixed anchorage’ thin film. Figure 1 shows the hypothesized reaction route for 1:1 equimolar mix and the reaction route the respective alkynes may undertake for both the hydrosilylation methods. The results may help understand how presence of initial surface radicals may direct the outcome as well as further examine the behavior of low temperature hydrosilylation setup.

### XPS surface analysis

**Figure 2:**
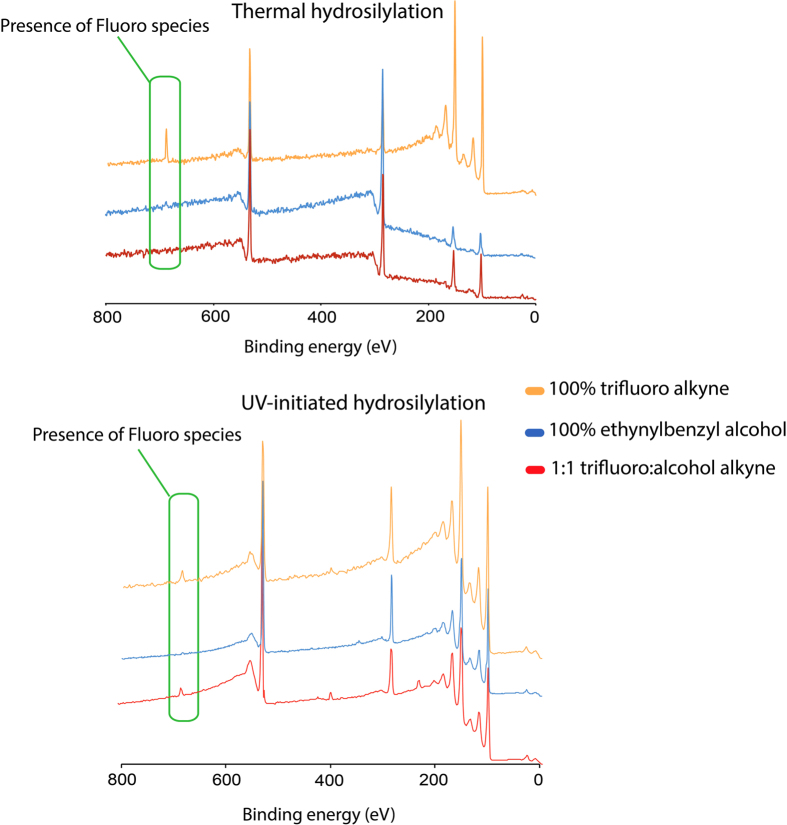


In this study, two sets of six chemical grafting on silicon (111) surfaces were prepared, three via thermal and other three using UV-initiated hydrosilylation. Each set of surfaces was chemically grafted in dilute concentrations of three different alkyne compositions, namely (1) 0.3 M of 100% trifluoroalkyne, (2) 0.3 M 100% ethynylbenzyl alcohol and (3) 0.3 M of a 1:1 equimolar mix of both. After the respective chemical grafting, XPS survey spectrum (Fig. 2) had revealed that under thermal hydrosilylation setup, fluorine F1s signal (688 eV) was only detectable for the 100% trifluoroalkyne modified surfaces but not on the 1:1 equimolar mix samples. On the contrary, for UV-initiated surfaces, F1s signals were detectable for both the 100% trifluoroalkyne modified surfaces as 1:1 equimolar mix surfaces. Atomic composition analysis on the various surfaces had also revealed several interesting observations (Table S1). Firstly, the percentage of fluorine on thermally grafted 100% trifluoroalkyne surfaces were observed to be at 4.74%, which was comparable to the values reported by UV initiated surfaces (3.31%) as shown in supplementary information S1. Based on the atomic composition of fluorine, the conclusion was that both thermal and UV hydrosilylation method for the trifluoroalkyne had yield relatively comparative fluoride percentages to the surface. However, the slightly elevated oxygen content (21.08%) for the UV-initiated hydrosilylation of the trifluoroalkyne grafted surfaces may suggest that oxidation from water could be a cause as residual water was not removed from the solvent at the lower reaction temperature compared to thermal hydrosilylation. Hence, this may had contributed to slightly higher oxidation and thus the increase in O1s signal on the surface for the 100% trifluoroalkyne. On the other hand, discerning the nature of O1s from ethynylbenzyl alcohol series from mere atomic composition data was not ideal, as the oxygen from the alcohol group would also contribute to the overall oxygen content on the film. Nonetheless, it was possible to see that the percentage of oxygen had reduced significantly in the UV-initiated hydrosilylation of the 1:1 equimolar mix (19.90%) as compared to the thermal hydrosilylation (21.58%) although it must be mentioned that the C1s content on the thermal hydrosilylation surfaces were also very high (53.06%) as compared to UV-initiated surfaces (29.41%).

**Figure 3:**
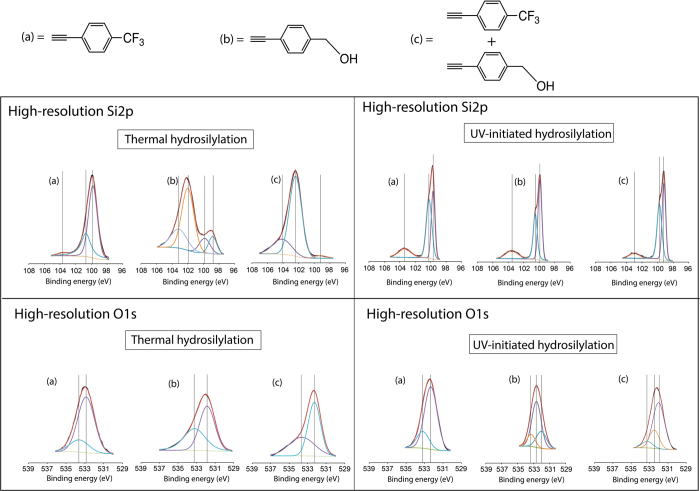
High resolution Si2p and O1s spectra of (**a**) 100% trifluoroalkyne, (**b**)100% ethynylbenzyl alcohol (**c**) 1:1 equimolar mix of both alkynes.

In order to identify the exact chemistry of the linkage (whether Si-C or Si-O-C), high resolution Si2p and the O1s scans were performed for all the surfaces. All peak positions and their respective assignments as well as the FWHM are listed in table S2 and S3. As illustrated in Fig. 3, high resolution Si2p (~103.0–104.2 eV) had revealed that the oxidation level for thermal hydrosilylation for the 100% trifluoroalkyne (Fig. 3a top row) was slightly lower than that from the UV initiation surface. Two core peaks at 99.4 eV and 100.5 eV were noticeable for the thermal reaction and were typically assigned to the Si2p_3/2_ and Si2p_1/2_ binding states respectively. Furthermore the nominal indicative position of Si-C typically situates at 99.8–100.5 eV^28^ and thus this may be attributed to the Si-C linkage although care must be taken during the assignment of this peak considering it overlaps with the Si2p_1/2_ (100 eV). This was also the case for the UV-initiated reactions for the trifluoroalkyne with the secondary core level peak at 100.2 eV.

On the 100% ethynylbenzyl alcohol grafted surfaces (Fig. 3b top row), an emerging major peak was observed centering at 102.0 eV for thermal hydrosilylation and this was attributed to the Si-O-C linkage as previously reported^21^,^28^,^29^. This was however not noticeable in the UV-initiated surface and the shaper secondary core level peak located at 100.5 eV may suggest that the surface predominantly underwent Si-C reaction.

In the 1:1 equimolar mix surfaces, thermal hydrosilylation (Fig. 3c, top row) had again shown the emergence of the Si-O-C linkage (102.3 eV) as well as the clear absence of this peak from the UV-initiated hydrosilylation while there was a significant reduction in the elemental Si core level peaks (99.3–100 eV). On the other hand, we were unable to confidently deconvolute the Si-O-C peak (~102.2 eV) to the UV-initiated surfaces. This suggested that surface radicals would preferential react to the alkyne end to form Si-C bond while under thermal reactions at temperatures lower than 150 °C the surface had a preference toward nucleophilic reaction to form Si-O-C linkage over the Si-C linkage, as previously reported by our group^21^. One interesting observation was that the nature of the Si2p and the Si2s signatures for the ethynylbenzyl alcohol in the survey spectrum that was evidently different in thermal reactions compared to the UV-initiated conditions. This was subsequently described as an artifact of surface roughness from the breaking of the silicon-silicon backbonding as illustrated in the AFM section.

High-resolution O1s was deemed a valuable means for describing the formation of the Si-O-C bonds and its reasons would validate the claims that thermal hydrosilylation in our setup had indeed driven the ethynylbenzyl alcohol to react at the OH end while UV initiated surface can proceed in both ends. As shown in Fig. 3a (bottom row), both thermal hydrosilylation of the trifluoroalkyne proceeded to form two core level peaks deconvoluted at 532.8 eV (Si-O) and 533.4 eV (Si-O_x_) while UV hydrosilylation yielded 532.3 eV (absorbed water)^30^ and 533.2 eV (Si-O_x_). In the ethynylbenzyl alcohol, as shown in Fig. 3b (bottom row), on the thermal functionalized surfaces, the main O1s peak were observed centered at 531.9 eV while two peaks for the UV functionalized surface was observable at both 532.0 and 532.7 eV. This latter peak at 532.7 eV was assigned to be the characteristic hydroxyl C-OH bond^31^,^32^,^33^, arising from the free end terminus of the ethynylbenzyl alcohol that was exposed to the surface in case Si-C bonding was formed from the alkyne end of the molecule. Describing the peak at 532.0 eV was more challenging. Firstly, in the arrangement of the Si-O-C bond, an oxygen atom is sandwiched between a silicon and a carbon atom and this may increase the overall electrostatic repulsion which would subsequently decrease the bonding energy, as was reported previously in literature^34^. This led us to conclude that the predominant oxygen species in these samples was associated with the Si-O-C linkage due to the downshift in binding energy. Considering that we observed only one 531.9 eV core level peak in the thermal hydrosilylation setup while we observed both the free C-OH (532.7 eV) and the 532.0 eV (Si-O-C) peaks in the UV-initiated surfaces, this had permitted for a logical speculation that the UV radicalized surface may have permitted for the formation of both Si-C and Si-O-C linkages simultaneously. The deconvoluted O1s spectra for the 1:1 equimolar mix (Fig. 3c, bottom row) had also revealed that Si-O-C was the predominant species on thermally and UV treated surface (532.1 and 532.0 eV respectively). While surface FTIR techniques to examine silicon oxide and monolayer profiling on thin film were often reported from literature^35^,^36^,^37^,^38^, previous studies as well as reports from the current authors, who had extensively used in the past FTIR (in an ATR geometry) to study (sub)monolayers grafted onto internal and external silicon surfaces, had shown the difficulties of discerning Si-O-C and Si-O-Si bonding on the surface^23^,^39^,^40^. Hence, surface analysis via FTIR, while commonly used in understanding grafting chemistry from literature, was not utilised in this paper.

**Figure 4:**
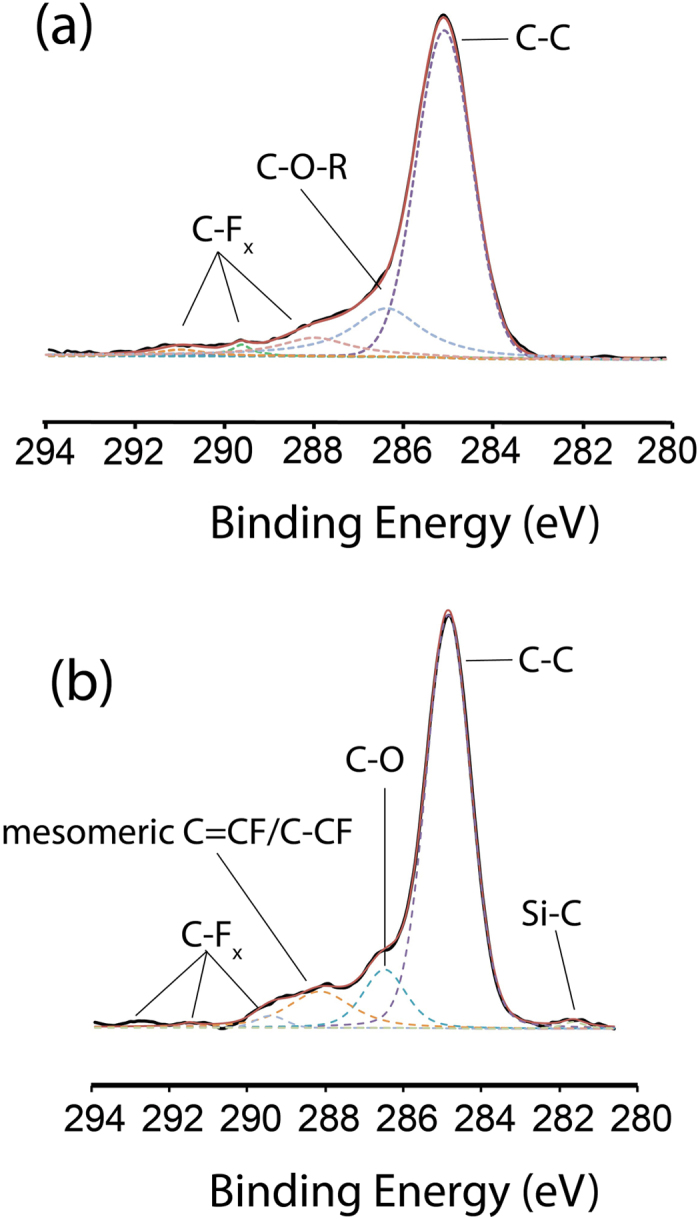
High-resolution XPS C1s spectra of (**a**) thermal hydrosilylation of the 1:1 equimolar mix and (**b**) UV initiated hydrosilylation of the 1:1 equimolar mix.

In high-resolution C1s spectra (Fig. 4) of the thermally treated 1:1 equimolar mix, apart from the nominal C-C bond at 285 eV, a broad peak centering at 286.2 eV was observed and this was indicative of the epoxy type C-O-R linkage as reported previously^13^,^41^. The broadness of that peak (FWHM = 2.6 eV) had seemingly suggested that the close proximity to the benzyl ring might have given rise to a variety of different C1s transition states of the C-OR and this may help explain the broad FWHM. Although trace amount of fluoride was detected (0.05%) from atomic concentration analysis, it was still possible to deconvolute the various C-F_x_ peaks (288.1 eV, 289.4 eV and 291.5 eV respectively^42^,^43^,^44^. In the thermal hydrosilylation, no observable Si-C peak (typically ~282.5–281.5 eV) could be fitted although a slight notch in signal around the region was noticeable. In the UV-initiated samples, the Si-C peak emerged at 281.5 eV (Fig. 4b) and this was the clearest evidence that UV-initiated hydrosilylation allowed alkynes to be grafted to the surface via Si-C linkage^45^. In fact, eliciting the Si-C of a monolayer from XPS analysis was not easy considering the many similar reports in literature that experience similar difficulties^46^. A broad C-O signal was detected at 286.3 eV and could either be assigned to the alcohol (functionalized with the ethynylbenzyl alcohol via the Si-C bond from the alkyne end) or to a small population of Si-O-C linked alkynes. What was most prominent was the stretch centering at 288.1 eV which was C*-CF bond that was previously reported in literature^47^ although the broadness of peak may suggest a mesomeric effect arising from the fluorocarbon situated next to a benzyl ring. . As both C-O and C*-CF was observable from the spectra for the UV initiated reaction (Fig. 4b), it was conceivable to suggest that on a radical-rich surface, reaction for the both selected alkynes could have proceeded either from the alkyne end or through the alcohol end.

Herein, the XPS results was in agreement with previously reported studies on functionalizing trifluoroalkynes at temperatures lower than 150°C^21^. The data suggested that in the absence of radicals on silicon surface, alkyne attachment (trifluoroalkyne) through the Si-C linkage to the silicon surface was primary driven by hydrogen abstraction in accordance to the kinetic model proposed by Horrocks *et al.*^22^. However the grafting of the alkyne to the surface via the triple bond end was complicated by the presence of the nucleophilic proneness from the alcohol group of ethynylbenzyl alkyne (in the 1:1 equimolar mix surfaces) that may have out-contested the trifluoroalkyne species to form covalent linkage (via Si-O-C bond) to the surface. On the other hand, in UV-initiated hydrosilylation setup, it was possible to observe the formation of a mixed monolayer, by which Si-O-C and Si-C linkage were indiscriminately formed although Si-C would be deem the prominent linkage based on the intensity of the peaks. Thus, the reactivity of the ethynylbenzyl alcohol had ‘switched’ between the surfaces and this could affect its surface wettability profile in the following section.

### AFM analysis

**Figure 5:**
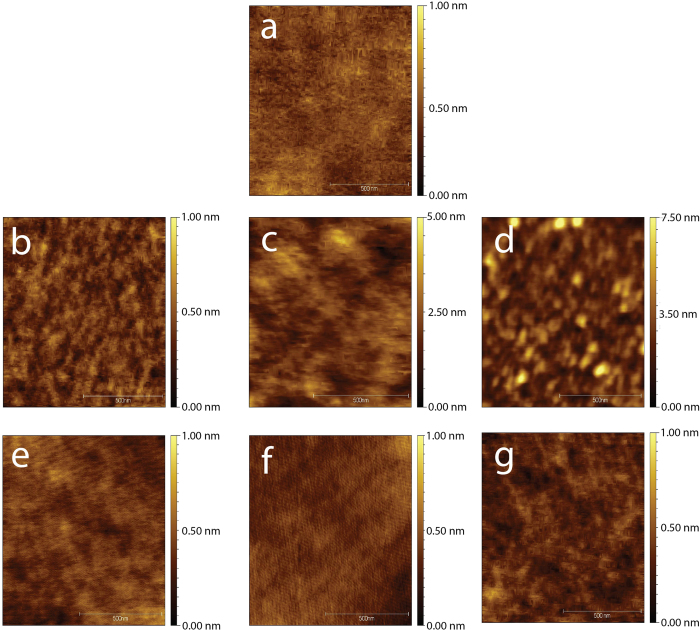
Atomic force microscopy of pristine Si (111) of the (**a**) pristine surface (**b**) thermal hydrosilylation of the trifluoroalkyne, (**c**) ethynylbenzyl alcohol and (**d**) 1:1 equimolar mix. Surfaces of the UV-initiated hydrosilylation was represented by (**e**) trifluoroalkyne, (**f**) ethynylbenzyl alcohol and (**g**) 1:1 equimolar mix. X and Y-axis of the images were 1 μm by 1 μm in dimension.

From our previous study, we noticed a roughening of the oxidized layer from angle resolved XPS (ARXPS) on the surface that had been passivated by the 100% ethynylbenzyl alcohol^21^. This could be explained if the breaking of silicon-silicon backbond may have intrinsically contributed to a roughening of the surface at the nanometric scale^21^. Further AFM analysis also revealed such a mild surface roughening (Fig. 5) arising from the alcohol species nucleophilic reaction to the surface, creating nanosized pits of 1–3 nm from cross sectional analysis. Root mean squares (rms) are tabulated in supplementary information table S4. Surface roughness was comparable in all samples with the exception of thermally treated surfaces with the presence of ethynylbenzyl alcohol, which showed an increase in roughness (0.96 nm ± 0.13 nm for thermally functionalized ethynylbenzyl alcohol and 1.08 nm ± 0.22 nm for the thermally functionalized 1:1 equimolar mix).

**Figure 6:**
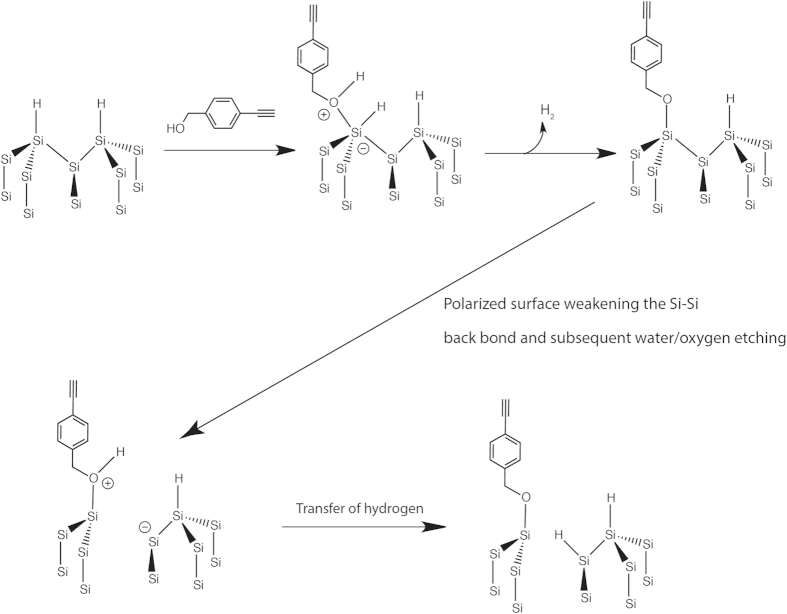


Clearly, the nucleophilic reaction to form Si-O-C induced by the presence of alcohol to the surface had resulted in a roughening effect on the silicon that subsequently raised its hydrophobicity profile. How the reaction actually occurred was presented in Fig. 6 in a reaction model that followed closely to the proposed mechanism by Cleland *et al.*^27^ and others^48^,^49^. Upon the interfacing of the OH with silicon hydride (Si-H), an intermediate of Si-O-R was first formed and subsequently with the ejection of hydrogen, the stable Si-O-C linkage was formed. However, based upon AFM analyses, we had shown that interaction with the alcohol resulted in a mild roughening of the surface in the thermal setup. This was highly unusual, considering that alcohol based reaction on Si-H surface does not typically exhibit surface etching effects as reported previously in literature^49^,^50^. There are two primary reasons that may explain why this may have happened, and both reasons were provided by the previous study performed by Boukherroub *et al.*^50^. Firstly, lengthy alcohol reaction time would render Si (111) surface more susceptible toward etching from alcohol species. Secondly, the presence of water/oxygen could well expedite the etching process. These two conditions were especially prominent in thermal hydrosilylation of 1:1 mix which yielded the highest rms values due to the prolong reaction time (18 h) as well as the collective influence of water and trace oxygen. However, in the thermal reaction of trifluoralkyne (where Si-C bonds were predominant), no surface etching was observed even though it was subjected to the same conditions. This was probably because Si-C linkage does not exhibit the same strength in polarity compared to Si-O-C bonds. This had led to our postulation that a change in polarity on the surface, such as a result of a Si-O-C bond, would have aid in water/oxygen etching and the gradual breaking of the Si-Si backbonds. Also, in the case of UV-initiated hydrosilylation of the ethynylbenzylalcohol as well as the 1:1 mix, the surface topography remained relatively smooth and this had suggested that the reaction time in the UV setup for the alkynes to form surface Si-C linkage was extremely rapid. Considering that Si-C linkages do not exert similar polarity effect like those from Si-O-C, residual water etching was less profound.

### Sessile droplet contact angle measurements

**Figure 7:**
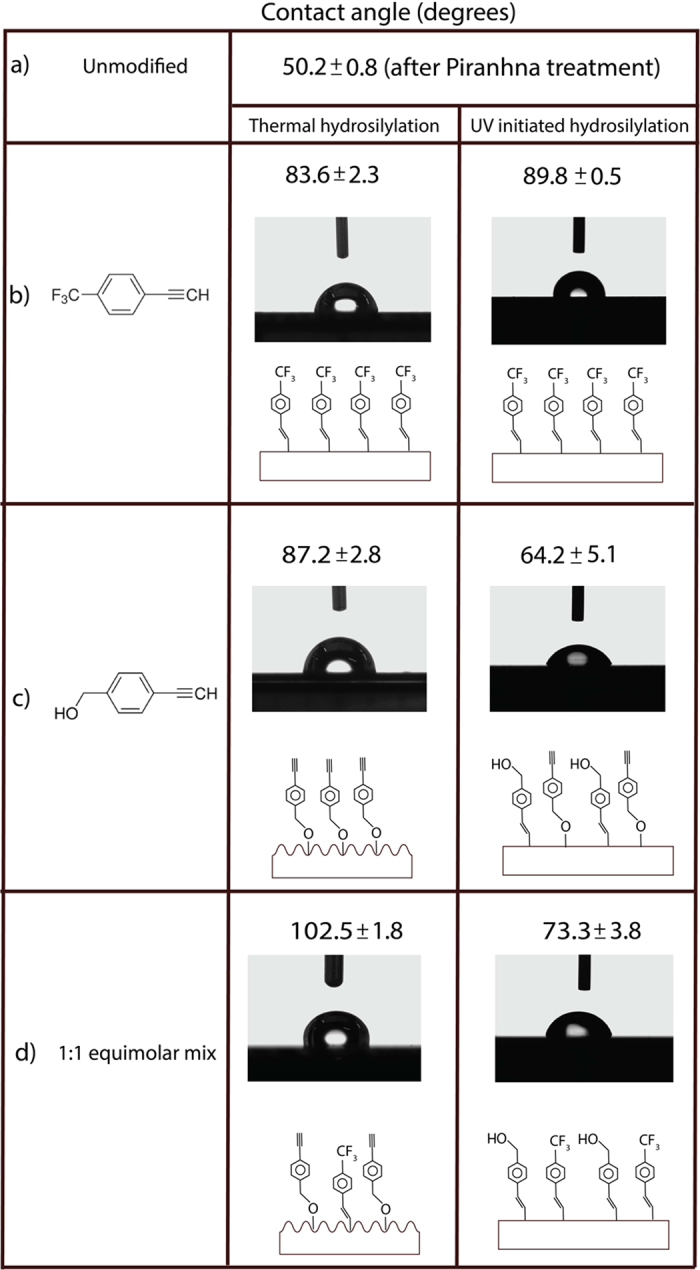
Sessile drop contact angle measurements of all surfaces treated under the different hydrosilylation methods. Note that the surface roughening coupled with Si-O-C linkage for the ethynylbenzyl alcohol had led to a increase in CA after thermal hydrosilylation while the surface had ‘switched’ back to Si-C bond formation in UV hydrosilylation, thus reducing the CA due to the exposed OH species.

A study of contact angle was also carried out in order to measure the wettability of these grafted monolayers. As shown in Fig. 7, both thermal and UV initiated hydrosilylation with 100% trifluoroalkyne produced surfaces of relatively similar wettability profile (83.6 ± 2.3° and 89.8 ± 0.5° respectively). Interestingly, on the 100% ethynylbenzyl alcohol surfaces, thermal hydrosilylation produced a surface that showed marginally higher contact angles (87.2 ± 2.8°) than those of 100% trifluoroalkyne. Considering that if the ethynylbenzyl alcohol was functionalized via the Si-C linkage, OH groups would be exposed to the surface in the case and this would inherently reduce the wettability. However, if the mode of reaction to the silicon hydride was nucleophilic, i.e. Si-O-C linkage, it was therefore possible to conceive that the exposed functional group was the opposite acetylene group, which could easily explain the rise in hydrophobicity. The CA values registered also marginally higher compared to previous report by Ciampi *et al.*^51^ with the acetylene termination although it must be stated that our silicon surface was of the (111) orientation rather than the reported (100) as reported, hence increasing the surface organics saturation. On the other hand, UV initiated reaction for the ethynylbenzyl alcohol had provided strikingly different wettability profile (64.2 ± 5.1°). This can only be explained by the formation of Si-C linkage that would expose the OH species to the surface thus increasing its hydrophilic, as shown in the inset in Fig. 5. Thus, the switch in binding chemistry could be observed between the two different functionalization techniques.

Based on Cassie-Baxter’s principle of wettability, these considerations help explain why the contact angle of a 100% ethynylbenzyl alcohol functionalized surface was much higher compared to those produced by 100% trifluoroalkyne. What was more notable was that under UV initiation, the surface wettability behaviour had completely reversed for the ethynylbenzyl alcohol (64.2 ± 5.1°) and this can only be described by having an exposed OH group as a result of the grafting. Thus it is not unreasonable to suggest that with existing radicals (UV initiated hydrosilylation) on the silicon surface, there was more motivation for the alkynes to form Si-C linkages. This observation was also reinforced by the contact angle results obtained by the experimental set of 1:1 equimolar by which thermal hydrosilylation produces a very high hydrophobicity value (102.5 ± 1.8°) while UV initiated surfaces recorded a substantially lower contact angle (73.3 ± 3.8°). In the scheme of things, the rms of our silicon wafer (0.09 nm) was found to be comparable to those as reported in literature (0.05–0.26 nm)^52^. However, what was most interesting was that we were able to obtain an extremely high hydrophobicity (102.5 ± 1.8°) from the thermal hydrosilylation of the 1:1 mix and considering that the rms registered was only 1.08 nm ± 0.22 nm, the surface is still relatively flat. The significance in this data was that our system was able to utilize extremely short alkyl molecules and was still able to obtain the comparably high CA values without the usage of long alkyl chains to attain the same effect^53^ on relatively flat surface. This high hydrophobicity values could be attributed to three factors: (1) the formation of the Si-O-C bond exposing the alkyne at the distal end, (2) the mild roughening of the surface from water/oxygen etching and (3) the presence of the trifluroalkyne in the film. On the other hand, the UV-initiation of the 1:1 mix had produced a surface with predominantly Si-C bonding and two distal functionalities would be present, the trifluoro and the hydroxyl. Therefore a reduction in CA was observed when compared to all 100% trifluoroalkyne reactions, either via thermal or UV hydrosilylation.

To further validate the point of our surface roughening values inducing the high hydrophobicity observed, atomically flat n-type silicon was also thermally grafted with 100% ethynylbenzyl alcohol and does not exhibit any topographical roughening on the surface due to the lack of hole carriers (see figure S1 in supplementary information). XPS examination of the atomically flat n-type also demonstrate the formation of Si-O-C bonding (see figure S2 in supplementary information) but the contact angle (see figure S3 in supplementary information) were substantially lower (70°) than compared to the alcohol grafted roughened surface (figure 5c) of the p-type measuring at 87°. Hence this shows that the roughening effect from the Si-O-C bond formation was responsible for the increment in hydrophobicity profile.

Following the above argument on XPS quantification of the atomic concentration and of the wettability data set, it was possible to draw the conclusions that (1) in low temperature hydrosilylation, in the presence of ethynylbenzyl alcohol, the surfaces predominantly underwent nucleophilic reaction to form Si-O-C linkage; (2) UV-initiated reactions does not give preference towards the nucleophilic reactions and the hydrosilylation resumes towards the formation of Si-C bonds; and (3) water may act as to destabilize the silicon-silicon back-bonding in the presence of a highly polarized Si-O-C monolayer.

## Conclusions

Bifunctional molecules are extremely important towards forming good quality monolayers on silicon, especially for bioactive purposes. In view of a wide range of grafted monolayers carrying OH groups in literatures, e.g. polyethylene glycol functionalization, findings from this study may help to address some fundamental issues, especially regarding thermal and UV based hydrosilylation techniques. Furthermore, the issue of residual water and oxygen in solution was also found to have a subtle effect on the overall chemistry of the linkage and material and this may prove to be important in designing good quality Si-O-R type films on silicon surface.

At temperature less than 150 °C where surface radicals were absent, the surface finds more energetically favourable to undergo nucleophilic reaction to form Si-O-C in the presence of OH carrying species. In the absence of OH carrying species, as in the case for the 100% trifluoroalkyne, Si-C was formed through the hydrogen abstraction model as proposed by Horrocks *et al.* However, when radicals were introduced at the onset of the reaction (UV initiated reaction), the surface switched back to favouring the formation of Si-C linkage regardless of the presence of alcohols.

It was found that the presence of Si-O-C linkage on the surface could induce enough polarity for the residual water present in the solution to assist in the breaking/weakening the silicon-silicon backbond, hence roughening the surface. This effect of surface roughening had subsequently given rise to a very different wettability profile on the surface.

## Methods and Materials

### Materials

Silicon wafer (111), boron-doped, had resistivity of 0.01-0.018 Ω cm and were used in this experiment. Sulfuric acid (Aldrich) and hydrogen peroxide (BDH Prolabo) were of semiconductor grade. 4-ethynylbenzyl alcohol and 4-Ethynyl-α,α,α-trifluorotoluene (trifluoro) were purchased from Sigma-Aldrich. All other chemicals, unless differently stated were used as received without further purification.

### Thermal Reaction Protocol

In a setup similar to Ciampi *et al.*^51^, Silicon wafers were cut into pieces (approximately 20 × 20 mm^2^) and cleaned for 30 min in hot Piranha solution (95 °C, 1 vol 33% aqueous hydrogen peroxide to 3 vol 95–97% sulfuric acid). Surface was then transferred to an aqueous 2.5% hydrofluoric acid for a duration of 90 seconds. Subsequently, the samples were transferred into sample of 4-Ethynyl-α,α,α-trifluorotoluene (0.3 M in Mesitylene) inside a custom-made Schlenk flask that had underwent a minimum of 20 freeze-pump-thaw cycles). The sample was kept under a stream of nitrogen and immersed in an oil bath set to 130 °C for 18 hours. The flask was then opened to retrieve the functionalized surfaces and were rinsed and sonicated in copious amounts of chloroform, ethyl acetate, and then ethanol before being analyzed.

For the 4-ethynylbenzyl alcohol-based layer, silicon surface was also functionalized in similar fashion and with the same molar concentration. Functionalized surface sample was rinsed consecutively with copious amounts of chloroform, ethyl acetate, and then ethanol before being analyzed.

In the third set of thermal reaction, a mixed solution of 0.3 M 4-ethynylbenzyl alcohol and 0.3 M 4-ethynyl-α,α,α-trifluorotoluene was co-solubilized in mesitylene and the solution underwent a minimum of 20 freeze-pump-thaw cycles to remove all oxygen. Subsequently, the hydrogen-terminated surface was introduced quickly into the solution inside the reaction vessel and the reaction was set to 130 °C for 18 h. The functionalized-surface sample was rinsed consecutively with copious amounts of chloroform, ethyl acetate, and then ethanol before being analyzed.

### UV initiated Hydrosilylation

Silicon wafers were pre-prepared in similar fashion to that in the thermal hydrosilylation protocol. Subsequently, the surfaces were transferred, taking extra care to completely exclude air from the fused silica reaction vessel (a custom-made fused silica flask), to a degassed (through a minimum of 20 freeze-pump-thaw cycles) sample of 4-Ethynyl-α,α,α-trifluorotoluene (0.3 M in Ethyl Acetate). 254 nm (4.88 eV) UV radiation was provided to the surface using a commercial 6 W Hg tube. The choice of custom-made quartz Schlenk flask made from fused silica ensures a very high transmittance of the 254 nm light to the sample, up to 90%.

The experimental setup is arranged with the UV lamp held in vertical position with adjustable distance from the reaction vessel, so that the UV light impinges perpendicularly to the sample surface and the power density can be easily varied. Vessel and lamp are enclosed in a dark box so that no light other than that from the UV lamp can reach the sample.

A calibration of the light intensity was performed using a large area calibrated Silicon photodiode from Hamamatsu photonics, showing that the experimental setup is capable to deliver from 1.2 mW/cm^2^ down to 100 μW/cm^2^ (lower values can be easily obtained inserting other filters) of 254 nm UV light intensity. The lamp-to-sample distance was adjusted in order to have 700 μW/cm^2^ power density. The surfaces were exposed to UV for 6 hours then rinsed consecutively with copious amounts of chloroform, ethyl acetate, and then ethanol before being analyzed.

The reaction was also performed for the 4-ethynylbenzyl alcohol at 0.3 M in ethyl acetate. In conjunction, a mixed solution of 0.3 M 4-ethynylbenzyl alcohol and 0.3 M 4-ethynyl-α,α,α-trifluorotoluene was co-solubilized in ethyl acetate and also subjected to UV-initiated hydrosilylation.

### Contact Angle Measurements

The water contact angle (CA) values were acquired on a Dataphysics OCA-20 goniometer setup at room temperature in ambient atmosphere. This instrument consists of a CCD video camera with a resolution of 768 × 576 pixel and could take up to 50 images per seconds. For each sessile droplet measurement three separate 5 μl droplets were dispensed onto the selected sample and the drop images were recorded. All drop images were then processed by an image analyser that calculated both the left and right contact angles from the droplet shape with an accuracy of ± 0.1°.

### Atomic Force Microscopy Measurements

Atomic force microscopy (AFM) images were acquired on a Hitachi SPA 300 HV Scanning Probe Microscope running an in-build AFM tapping mode with Tap 150Al-G model cantilever (Freq. 150 kHz, Force 5 N/m) in triplicates. Scan area on the surfaces were of 1 μm x 1 μm and the scan speed was set at 0.8 hz with the integral and proportional gain set at automatic mode. Post image processing was performed with Gwyddion MacOS version 2.38.

### X-ray photoelectron spectroscopy (XPS)

The XPS wide scan spectra were acquired using AXIS Ultra DLD, Kratos, equipped with an Al Kα X-ray source (1486.6 eV) at 10 mA, 15 kV, analyzing a 300 μm X 700 μm area under 3.9 × 10^−9^  Torr ultra vacuum environment inside sample analyze chamber. Analyses were performed in the hybrid lens mode with the slot aperture and the pass energy of the hemispherical analyzer set at 100 eV for the survey scan. Spectra were also obtained for the C1s, F1s, Si2p and O1s in high resolution for all samples. The spectra were subsequently analyzed using in-built Kratos Vision 1.5 software, which included with vision manager and vision processing.

## Additional Information

**How to cite this article**: Khung, Y. L. *et al.* Thermal and UV Hydrosilylation of Alcohol-Based Bifunctional Alkynes on Si (111) surfaces: How surface radicals may influence surface bond formation. *Sci. Rep.* **5**, 11299; doi: 10.1038/srep11299 (2015).

## Supplementary Material

Supplementary Information
