# Supplementary material for: Thermal and UV Hydrosilylation of Alcohol-Based Bifunctional Alkynes on Si (111) surfaces: How surface radicals influence surface bond formation
Source: Sci Rep. 2015 Jun 12;5:11299. doi: 10.1038/srep11299 (PMC4650888; doi:10.1038/srep11299)
Supplement: Supplementary Information [file srep11299-s1.doc]

Thermal and UV Hydrosilylation of Alcohol-Based Bifunctional Alkynes on Si (111) surfaces: How surface radicals influence surface bond formation

**Y. L Khung*^1,2*^*, S. H. Ngalim*^2^*, A. Scaccabarozi*^1^* and D. Narducci*^1^***

**Supplementary Information**


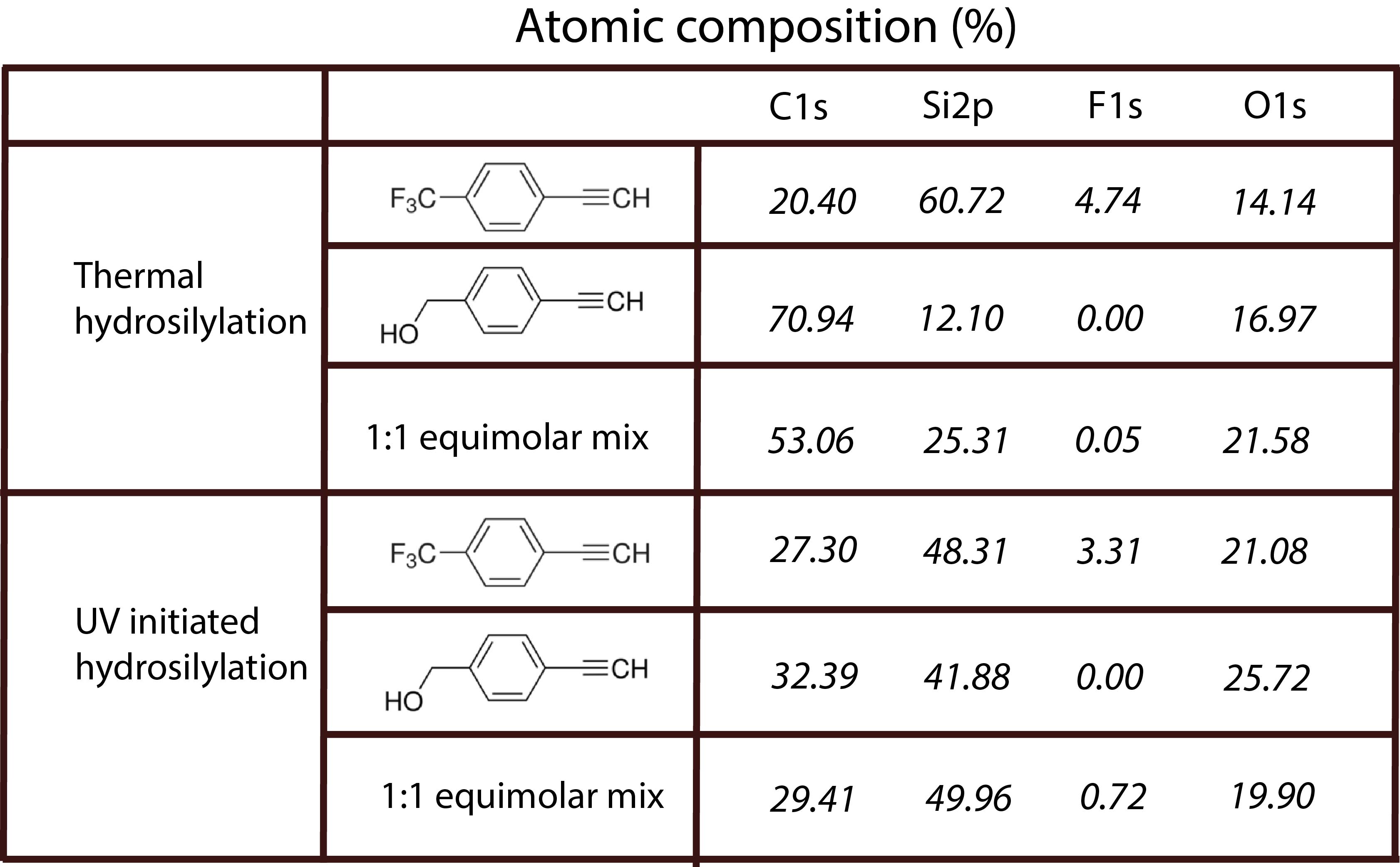


**Table S1. Atomic concentration (%) of all the respective surface grafting**

| **Thermal hydrosilylation** | **Peak (eV)** | | **FWHM** | **Area** | **Assignments** |
| --- | --- | --- | --- | --- | --- |
| Trifluoroalkyne | 99.4 | | 1.28 | 42168.2 | Si-Si (Si2p_3/2_) |
|  | 100.5 | | 1.69 | 20390 | Si-C |
|  | 103.6 | | 1.16 | 2128.5 | Si-O_x_ |
|  |  | |  |  |  |
| Ethynylbenyzyl alcohol | 99.1 | | 1.02 | 807.63 | Si-Si(Si2p_3/2_) |
|  | 100.1 | | 1.5 | 1129.42 | Si-Si(Si2p_1/2_) |
|  | 102.0 | | 1.8 | 6046 | Si-O-C |
|  | 103.2 | | 2.11 | 2276 | Si-O_x_ |
|  |  | |  |  |  |
| 1:1 equimolar mix | 99.3 | | 1.19 | 83 | Si-Si |
|  | 102.3 | | 1.66 | 8013 | Si-O-C |
|  | 103.3 | | 2.28 | 3279 | Si-O_x_ |
|  |  | |  |  |  |
| **UV-initiated hydrosilylation** | | **Peak (eV)** | **FWHM** | **Area** | **Assignments** |
| Trifluoroalkyne | 99.6 | | 0.55 | 5516 | Si-Si(Si2p_3/2_) |
|  | 100.2 | | 0.85 | 7551 | Si-C/Si-Si(Si2p_1/2_) |
|  | 103.4 | | 1.89 | 2203 | Si-O_x_ |
|  |  | |  |  |  |
| Ethynylbenyzyl alcohol | 99.8 | | 0.54 | 8289 | Si-Si(Si2p_3/2_) |
|  | 100.5 | | 0.64 | 6287 | Si-C/Si-Si(Si2p_1/2_) |
|  | 103.5 | | 1.84 | 2748 | Si-O_x_ |
| 1:1 equimolar mix | 99.2 | | 0.55 | 2838 | Si-Si(Si2p_3/2_) |
|  | 99.8 | | 0.73 | 2865 | Si-C/Si-Si(Si2p_1/2_) |
|  | 103.1 | | 1.54 | 527 | Si-O_x_ |

**Table S2. High resolution Si2p peak positions and assignments for thermal and UV-initiated hydrosilylation**

| **Thermal hydrosilylation** | **Peak (eV)** | | **FWHM** | **Area** | **Assignments** |
| --- | --- | --- | --- | --- | --- |
| Trifluoroalkyne | 532.8 | | 2.02 | 36261 | Si-O |
|  | 533.4 | | 2.69 | 15870 | Si-O_x_ |
|  |  | |  |  |  |
| Ethynylbenyzyl alcohol | 531.9 | | 2.07 | 31647 | C-O-R |
|  | 533.4 | | 2.92 | 17031 | Si-O_x_ |
|  |  | |  |  |  |
| 1:1 equimolar mix | 532.2 | | 1.68 | 28237 | C-O-R |
|  | 533.9 | | 2.86 | 11965 | Si-O_x_ |
|  |  | |  |  |  |
|  |  | |  |  |  |
| **UV-initiated hydrosilylation** | | **Peak (eV)** | **FWHM** | **Area** | **Assignments** |
| Trifluoroalkyne | 532.3 | | 1.62 | 2928 | Si-O-Si |
|  | 533.2 | | 1.56 | 751 | Si-O_x_ |
|  |  | |  |  |  |
| Ethynylbenyzyl alcohol | 532.0 | | 1.49 | 851 | C-O-R |
|  | 532.6 | | 1.37 | 2181 | Si-O/C-O-H |
|  | 533.3 | | 1.17 | 483 | Si-O_x_ |
| 1:1 equimolar mix | 532 | | 1.48 | 4720 | C-O-R |
|  | 532.5 | | 1.25 | 1529 | Si-O/C-O-H |
|  | 533.3 | | 1.32 | 622 | Si-O_x_ |

**Table S3. High-resolution O1s peak positions and assignments for thermal and UV-initiated hydrosilylated surface**

|  |  | |  | |
| --- | --- | --- | --- | --- |
| **Surface roughness (RMS)** | |  | | |
|  | **Thermal hydrosilylation** | | **UV-initiated Hydrosilylation** |  |
| Trifluoroalkyne | 0.17 nm ± 0.08 nm | | 0.09 nm ± 0.04 nm |  |
|  |  | |  |  |
| Ethynylbenzyl alcohol | 0.96 nm ± 0.13 nm | | 0.08 nm ± 0.02 nm |  |
|  |  | |  |  |
| 1:1 equimolar mix | 1.08 nm ± 0.22 nm | | 0.09 nm ± 0.05 nm |  |
|  |  | |  |  |
| Prestine silicon | 0.09 nm ± 0.05 nm | |  | |

**Table S4. Surface roughness (RMS) of the thermal and UV treated samples**


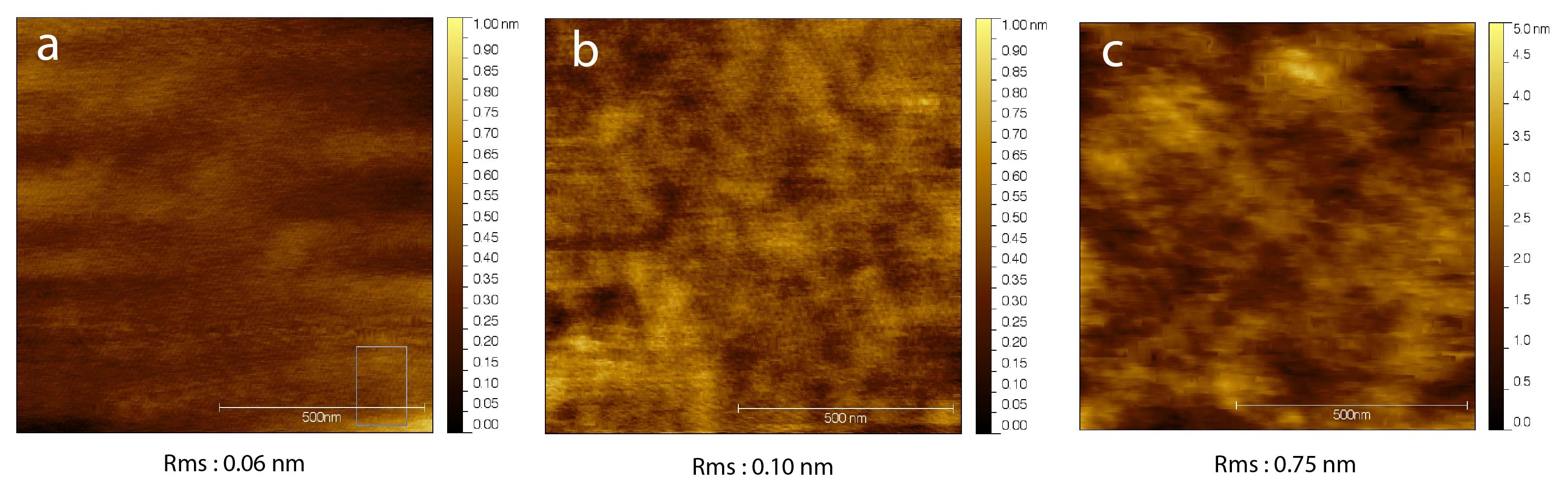
**Figure S1. (a) NH_4_F etched n-type silicon (111) surface, (b) thermal grafting of 4-ethynylbenzyl alcohol of the NH_4_F etched N-type silicon (111) surface**


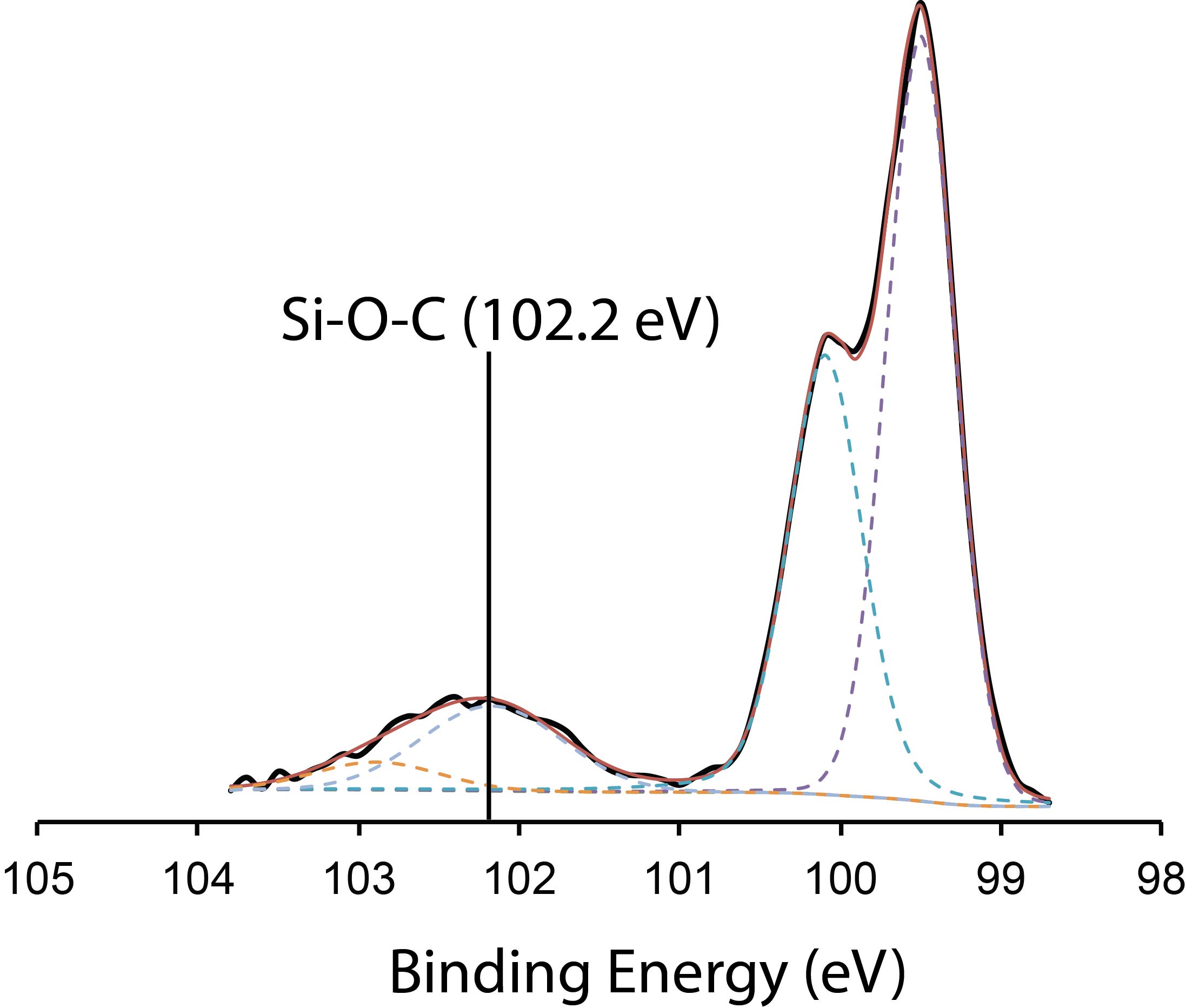


**Figure S2. XPS Si2p spectra of thermally grafted alcohol on atomically flat N-type silicon, exhibiting the same Si-O-C linkage as reported**


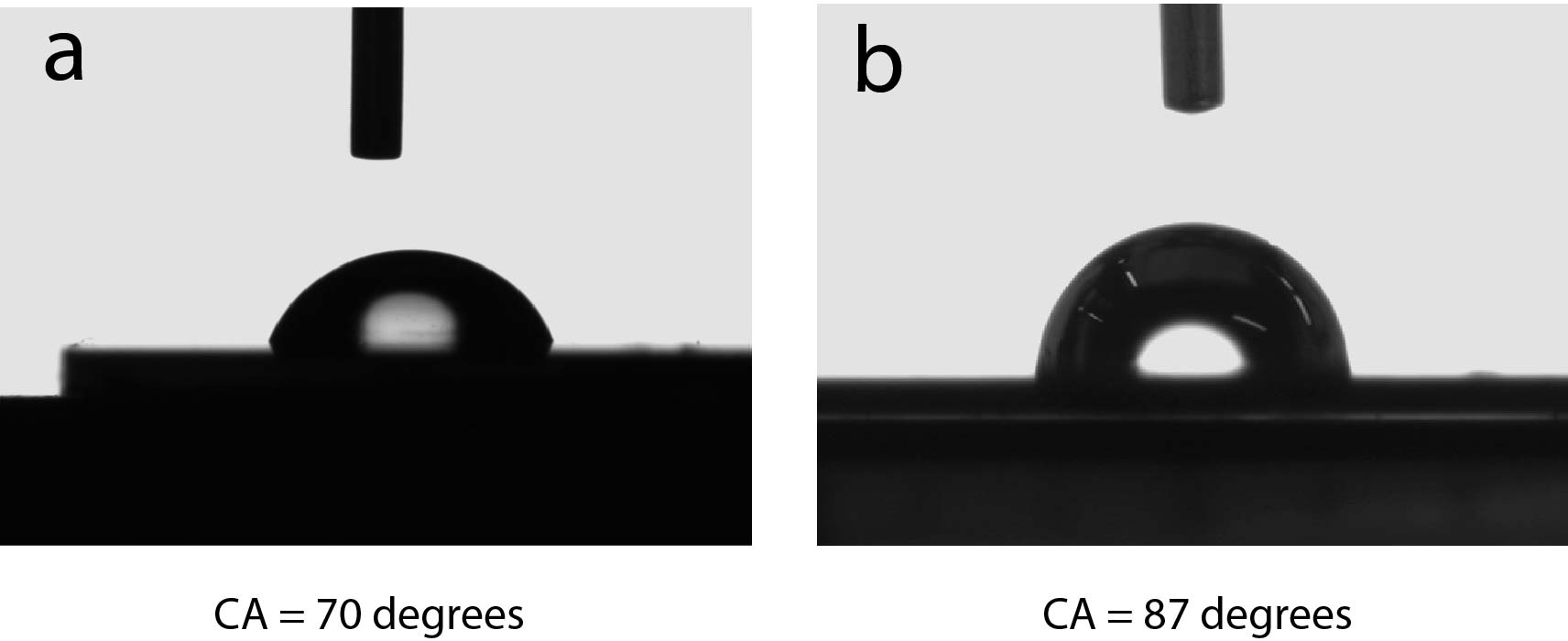


**Figure S3. Contact angle measurement on (a) thermally grafted alcohol on n-type atomically flat Si (111) and (b) thermally grafted alcohol alkyne on p-type Si(111) surface**
